# Copper and Copper/Zn Ratio in a Series of Children with Chronic Diseases: A Cross-Sectional Study

**DOI:** 10.3390/nu13103578

**Published:** 2021-10-13

**Authors:** Marlene Fabiola Escobedo-Monge, Enrique Barrado, Joaquín Parodi-Román, María Antonieta Escobedo-Monge, María Carmen Torres-Hinojal, José Manuel Marugán-Miguelsanz

**Affiliations:** 1 Faculty of Medicine, Valladolid University, Avenida Ramón y Cajal, 7, 47005 Valladolid, Spain; mctorresh@telefonica.net; 2 Department of Analytical Chemistry, Science Faculty, Valladolid University, Campus Miguel Delibes, Calle Paseo de Belén, 7, 47011 Valladolid, Spain; ebarrado@qa.uva.es; 3 Science Faculty, Cadiz University, Paseo de Carlos III, 28, 11003 Cádiz, Spain; joaquin_parodi@yahoo.es; 4 Department of Chemistry, Science Faculty, University of Burgos, Plaza Misael Bañuelos sn, 09001 Burgos, Spain; anto@ubu.es; 5 Department of Pediatrics of the Faculty of Medicine, Valladolid University, Avenida Ramón y Cajal, 7, 47005 Valladolid, Spain; jmmarugan@telefonica.net; 6 Section of Gastroenterology and Pediatric Nutrition, University Clinical Hospital of Valladolid, Avenida Ramón y Cajal, 3, 47003 Valladolid, Spain

**Keywords:** hypocupremia, hypercupremia, inflammatory response, risk of zinc deficiency, serum zinc/copper ratio

## Abstract

Copper is an essential micronutrient for humans. A cross-sectional and comparative study was done to assess serum Cu levels and serum copper/zinc (Cu/Zn) ratio and its association with nutritional indicators in a series of children and adolescents with chronic diseases. Anthropometric, biochemical, dietary, body composition, and bone densitometry assessments were carried out. Serum Cu and Zn were measured by atomic absorption spectrophotometry. Seventy-eight patients (55% women) participated. The mean serum Cu in the entire series and by nutritional status through body mass index (BMI) was normal. Serum Cu decreased significantly with age and was meaningfully higher in children than in adolescents. The risk of finding altered Cu levels in children and men was higher than in adolescents and women, respectively. Twenty-two per cent of patients had abnormal serum copper levels, 13 had hypercupremia, and four had hypocupremia. The Cu/Zn ratio was greater than 1.00 for 87% of the patients, which is an indicator of an inflammatory state. All patients with hypozincemia and hypocupremia had deficient Zn intake, but only 65% of the patients with hypercupremia had dietary Zn deficiency. Consequently, the Cu/Zn ratio could indicate an inflammatory state and a high risk of zinc deficiency in this specific child population.

## 1. Introduction

It is generally recognized that numerous chronic diseases have their origins in childhood [1]. Advances in the early diagnosis and treatment of illnesses have led to an increase in the prevalence of chronic disease in children and adolescents [2]. Although chronic diseases are rare in these age groups, they affect a non-negligible percentage of 10 to 20% of the child population [3]. Currently, more than 90% of children with chronic diseases or disabilities survive beyond the second decade, and more than 30% of youth ages 10 to 17 have a chronic illness [4]. The scope of the problem is highlighted by the childhood obesity epidemic leading to an escalation in the number of other chronic conditions [5]. Non-communicable diseases (NCD) are increasingly common causes of childhood illness and death [6]. The onset, symptoms, and evolution of chronic pathologies depend on the genetic expression and antioxidant–anti-inflammatory system of the organism, which in turn depend on the basic nutrients and their active forms [5], as occurs with copper.

Copper is an essential nutrient, which is present in almost every cell of the body [7] and involved in many functions [8]. It is an important catalyst of enzymes [9] that take part in oxidoreductions of, inter alia, lysyl oxidase and copper-zinc superoxide dismutase (SOD) [10]. Its functions are mainly related to the formation of connective tissue, iron metabolism, the development of the central nervous system, and cardiovascular functions (cholesterol metabolism) [11]. Copper increases as an acute-phase response in a variety of infections and inflammatory conditions [12]. Although Laine et al. (2020), in middle-aged and older men, suggested that the serum Cu level alone might be a better marker for future risk of an infection [13], other authors believe that the Cu/Zn ratio is a well-established feature of infections [14], including parasitic infections, such as Schistosoma mansoni, Enterobious vermicularis, and Trypanosoma cruzi infections and in giardiasis [15] or amebiasis [15,16] and tuberculosis (TB) [14,17]. The significantly higher Cu/Zn ratio at baseline in giardiasis and amebiasis [16], malaria [18], and TB [19] returned to normal after therapy. Furthermore, this Cu/Zn ratio has a diagnostic value in several human disorders [14]. 

The Cu/Zn ratio is between measurements of both Zn and Cu alone; the only one that may be associated with a reduced ability to maintain or regain homeostasis after a destabilizing event [20]. This ratio is mainly associated with inflammatory mediators rather than nutritional factors [21]. A high Cu/Zn ratio has been associated with chronic inflammatory diseases [22], malnutrition [20], increased oxidative stress, inflammation, and disrupted immune status in patients with chronic disease [23]. Albeit Strain described the pathophysiologic role of Cu in chronic disease in adults [24], information on serum Cu levels in chronically ill children and adolescents is scarce. For this reason, we hypothesized whether an abnormal serum Cu level is prevalent in a series of chronically ill children, or the Cu/Zn ratio might provide a stronger marker of Zn deficiency than either of the values alone. Thus, the purpose of this study was assessing serum Cu levels and the Cu/Zn ratio and its association with nutritional indicators in a series of children with chronic diseases. This research improves the existing literature in several ways. It would be the first study to explore this aspect in chronically ill children and adolescents. The zinc nutritional status of this series in specific patients was previously published [25]. 

## 2. Materials and Methods 

### 2.1. Study Site, Design, and Participants

**Figure 1:**
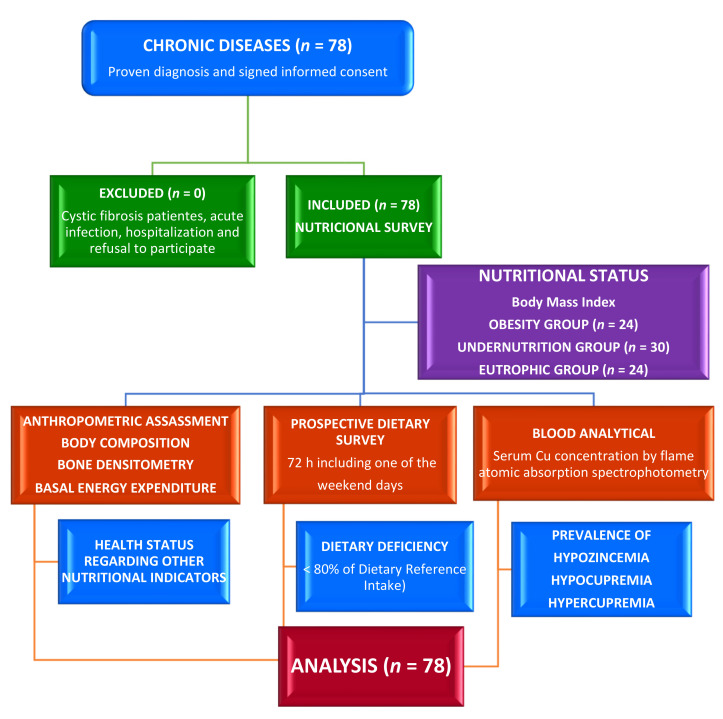
Flow diagram assignment of patients with chronic disease (*n* = 78).

The design of this cross-sectional and comparative study (Figure 1) to evaluate serum Cu and Cu/Zn ratio were previously described in these patients, assessing the nutritional zinc status through its intake and serum levels [25]. It was carried out in the Nutrition Unit of the Pediatrics Service at the University Clinical Hospital in Valladolid, Spain. The number of participants seen during the 18 months of the study determined the sample size. The inclusion criteria were children under 19 years of age with proven diagnosis of chronic illness. Chronic diseases include malnutrition of unknown cause, syndromic diseases, encephalopathies, kidney disease, hyperlipidemia, insulin-dependent diabetes mellitus, and eating disorders. Participants were classified by nutritional status into eutrophic, obesity, and undernutrition groups using BMI. Cystic fibrosis (CF) patients [26,27], acute infection, hospitalization, and refusal to take part were exclusion criteria. The time of chronic diseases was shown in months.

### 2.2. Ethical Consideration

The study protocol was reviewed and approved by the local ethics committee at the University Clinical Hospital (INSALUD-Valladolid, 14 February 2002), and was carried out in accordance with the Declaration of Helsinki. Written, informed consent was obtained from the relatives/guardians of all patients before taking part in this study.

### 2.3. Assessment of Phenotypical Characteristics

Data on age and gender were collected using questionnaires. An anthropometric evaluation of weight, height, and wrist, hip, waist, and mid-arm circumference was carried out using standard techniques. Z-score of weight-for-age, height-for-age, age-for-50° height or height age, weight-for-height, BMI-for-age, and BMI-height-age, and the mid-arm muscle area, fat-free mass, and fat mass were calculated using Frisancho [28] and Orbegozo tables [29]. Triceps, biceps, subscapular, and suprailiac skinfold were measured by standard methods with a Holtain Skinfold Caliper. Body composition was measured by anthropometry and bioelectrical impedance analysis (BIA) [RJL BIA-101 (RJL System, Detroit, MI, USA)]. Bone densitometry by ultrasound [DBM Sonic 1200 IGEA (Emsor S.A., Madrid, Spain)] was measured by the bone conduction speed (BCS) of the last four fingers of the non-dominant hand [30]. Basal energy expenditure (EE) or resting EE (REE) was measured by fasting indirect Calorimetry (IC) with a canopy system in standardized conditions [Deltatrac II (Datex-Ohmeda. Helsinki, Finland)].

### 2.4. Dietary Assessment

Participants were trained to register all the food that was consumed and the amounts according to household measurements. Analysis of reported daily intake of energy; fiber; carbohydrates; protein; lipids; monounsaturated, polyunsaturated, and saturated fats; vitamins A, B1, B2, B6, B12, C, D, E, niacin, and folic acid; and calcium (Ca), magnesium (Mg), iron (Fe), Zn, and iodine (I) were calculated from the food consumption records of a 72-h prospective dietary survey (including one of the weekend days), the week before of the blood test. Nutrient sufficiency was assessed using percentage of Dietary Reference Intake (%DRI) or adequate intake using the Mataix Food and Health software, which provided the percentage of actual nutrient intakes with respect to Spanish recommendations [31,32]. Less than 80%DRI was the cutoff used to categorize a dietary intake as inadequate. In this series, no patient had taken micronutrient and vitamin supplements. 

### 2.5. Clinical Evaluation

During the evaluation of each patient, in addition to assessing the clinical and nutritional status, neurological symptoms due to Cu deficiency were assessed, such as the presence or absence of dysarthria, rigidity, poor handwriting, tremor [33], gait difficulties (sensory ataxia), paresthesias in the upper and lower extremities, depression of distal reflexes, and distribution of overlapping sensory alterations in glove and sock (sensory/motor neuropathy) [34]. We evaluated whether the patients had diarrhea and the presence of some skin lesions related to zinc deficiency, such as hyperpigmented skin, rough skin, keratosis/keratitis, dermatitis, bullous/pustular dermatitis, and alopecia [35].

### 2.6. Laboratory Exploration

Fasting blood samples were collected, and serum was transported to the Laboratory of Instrumental Techniques of the Chemistry Department of the Valladolid University. To avoid bias, all serum samples, previously stored at −18 °C, were slowly thawed and then diluted (1:4) in deionized and demineralized water. Calibration curves (between 0 and 5 µg/dL) were made from aqueous solutions of the standards, using a wavelength of 324.8 nm, an analysis time of 4 s, an acetylene flow of 0.8 L/min, with a 0.5-nm slit, and a 4.5-mA lamp intensity. Calibration was carried out in mg/L. All the material was previously washed with 20% nitric acid and washed with deionized water. Serum Cu levels were analyzed by flame atomic absorption spectrophotometry (model PU9400 Philips) [36]. Less than 70 µg/dL and more than 140 µg/dL are the cutoffs used to categorize hypocupremia (Cu deficiency) and hypercupremia, respectively [37]. The Cu/Zn ratio [38] derived by calculation was evaluated as an alternative biomarker to assess the inflammatory and nutritional status and adverse clinical outcomes [39], where its normal obtaining values range between 0.7 to 1.0 [40]. The zinc/copper (Zn/Cu) ratio < 4.0 is often associated with an increase of the susceptibility to bacterial and viral infections [41]. Blood count, complete biochemical analysis, and the activity of acute-phase proteins, including C-reactive protein (CRP) > 4 U/L and erythrocyte sedimentation rate (ESR) in women > 20 mm/h and men > 15 mm/h, were measured using standardized methods. We evaluated the serum levels of folic acid; beta-carotene; vitamins B12, C, D, E, Ca, phosphorus (P), Mg, and Fe; total immunoglobulin (Ig) G, IgG1–4, IgA, IgM, and IgE; C3 and C4 complement; CD3, CD4, CD8, CD16 + 56, CD19 lymphocytes and CD4/CD8 ratio; and Insulin-like growth factor-1 (IGF-1) and insulin-like growth factor-binding protein 3 (IGFBP3). Serum prealbumin ≤ 18 mg/dL, albumin ≤ 3.5 g/dL as visceral protein reserve, transferrin ≤ 200 mg/dL, lymphocytes < 2000 cell/mm^3^, total cholesterol (TC) > 200 (mild-moderate risk) and >225 mg/dL (high risk), and low-density-lipoprotein cholesterol (LDL-C) > 115 (mild-moderate risk) and >135 mg/dL (high risk) were used as cutoffs to evaluate abnormal values.

### 2.7. Statistical Analysis

A database was created to analyze the results. The main variables studied were the serum Cu level and Cu/Zn and Zn/Cu ratios. Anthropometric, biochemical, dietary, body composition, bone densitometry, and basal energy expenditure were secondary variables. The distribution of anthropometric results (quantitatively and Z-scores) and biochemical data were described as mean, median, quartiles, standard deviation (SD), and range. The normal distribution of values was evaluated using the Kolmogorov–Smirnov test. Two-tailed Student t-test was used for unpaired or paired variables, and one-way analysis of variance (ANOVA test) and Pearson’s bivariate correlation test were used for normally distributed values. Categorical data were evaluated by Pearson’s Chi-square test (X^2^) with Yates’s correction and Fisher’s exact test (FET). A non-parametric test was used for the variables with non-normal distribution. Odds ratios (OR) were calculated to estimate the magnitude of the association between exposure and disease. Simple and multiple linear regression analyses were calculated to study the significant associations between two and more meaningful correlations. The IBM SPSS software version 24.0 (IBM Corp., Armonk, NY, USA) was used to carry out the statistical analysis. The significance level was established at *p* < 0.05 * and <0.01 **.

## 3. Results

The outcome of the anthropometric, dietary, biochemical evaluation, serum Zn levels, and clinical manifestations of hypozincemia in these patients had already been published [25]. Table 1 summarizes the basic characteristics of the children and adolescents in the entire series according to nutritional status via BMI. Seventy-eight patients (43 females, 55%) participated in this study. Ninety-nine percent of these patients were from Valladolid, 96% were Caucasian, and 4% were Romani. The average age was 9.6 ± 4.8 years old with median 10 and the range was 1–19 years. Forty-two patients (54%) were children, and 36 (46%) were adolescents. Serum Cu (*p* = 0.823) and Zn (*p* = 0.393), dietary Zn intake (*p* = 0.100), and Cu/Zn ratio (*p* = 0.423) had a normal distribution curve (Kolmogorov–Smirnov). 

In the whole series and according to nutritional status, the mean serum Cu and Zn, and the Zn/Cu ratio were normal, but the Cu/Zn ratio was high. Seventeen patients (22%) had abnormal serum copper, four of them had hypocupremia (5%), and 13 had hypercupremia (17%). Five patients had hypozincemia (6%). Sixty-eight participants (87%) had Cu/Zn ratio > 1.0, and four patients (5%) had Cu/Zn ratio > 2.0. Only one case had Zn/Cu ratio > 4.0. Although the mean serum Cu in the undernutrition group (114 μg/dL) was lower than in the eutrophic (122 μg/dL) and obese patients (119 μg/dL), this difference was not significant. Eight of the 21 malnourished children and one of the four eutrophic patients had lower weight for their age and abnormal serum Cu. There were no significant differences in gender or in the length of illness between the nutritional groups or in the Cu/Zn and Zn/Cu ratios according to gender and nutritional status. 

Table 2 lists all the patients with abnormal serum Cu and Zn, high Cu/Zn and Zn/Cu ratios, and deficient Zn intake. No case with hypocupremia was found in the eutrophic group. All the patients with hypozincemia and hypercupremia had higher Cu/Zn ratio > 1.0, but in children with hypocupremia this ratio was normal. Three patients with hypercupremia (23%) had a Cu/Zn ratio > 2.0. Only one 3-year-old eutrophic boy (8%) with deficient Zn intake, hypercupremia, hypozincemia, and high ERS had a Cu/Zn ratio > 2.0. Another malnourished 2-year-old boy (25%) had dietary Zn deficiency, hypocupremia, and a high Zn/Cu ratio. Among the hypocupremic patients, a 2-year-old boy with mitochondrial neuropathy and severe neurological involvement had a very low serum copper concentration (20 μg/dL) with normal serum zinc (117 μg/dL). Another male 15 year old with the severe malabsorptive syndrome had a history of posterior cord peripheral neuropathy. The other two female patients had no neurological symptoms. Only one child with obesity and hypozincemia had rough skin. Sixty-four percent of the patients had marginal zinc deficiency. Although all hypozincemic and hypocupremic patients had dietary Zn deficiency, only 65% of hypercupremic patients had deficient Zn intake.

**Figure 2:**
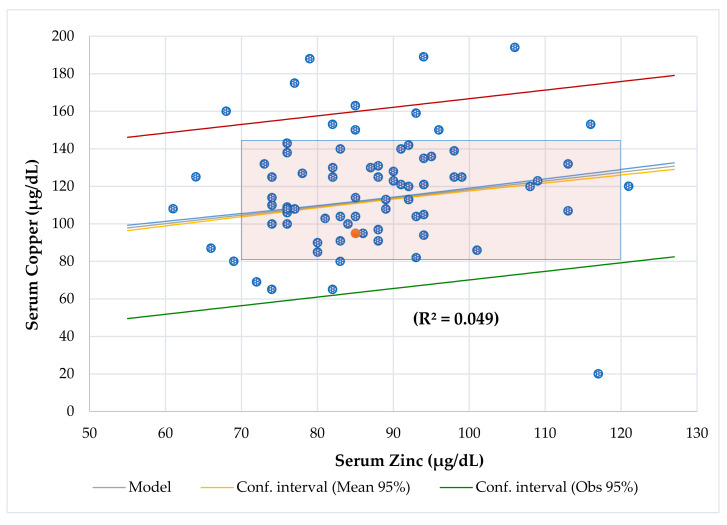
Regression serum copper (70–140 µg/dL) by zinc (70–120 µg/dL) adjusted for age and cutoffs.

**Figure 3:**
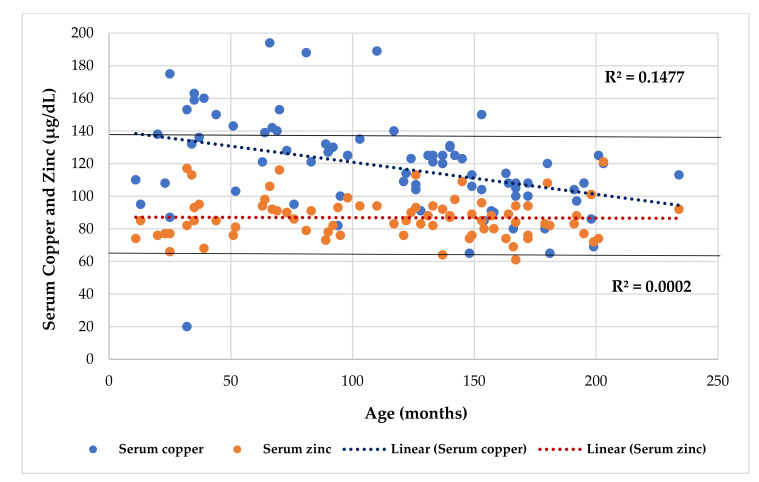
Regression serum copper and zinc (µg/dL) by age (months).

**Figure 4:**
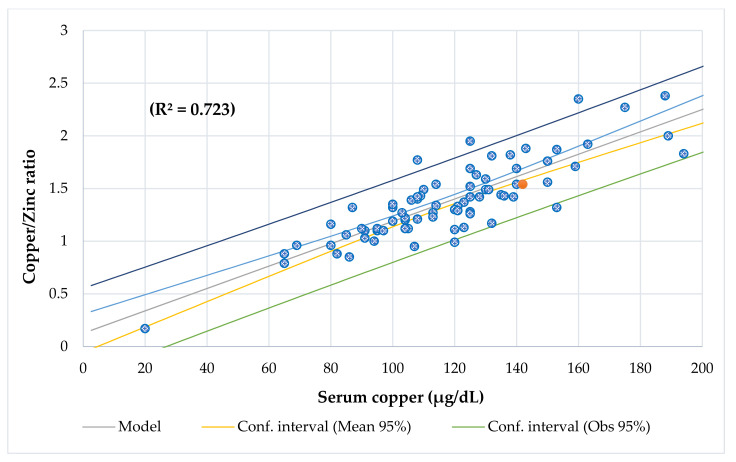
Regression Copper/Zinc ratio by serum copper (µg/dL).

**Figure 5:**
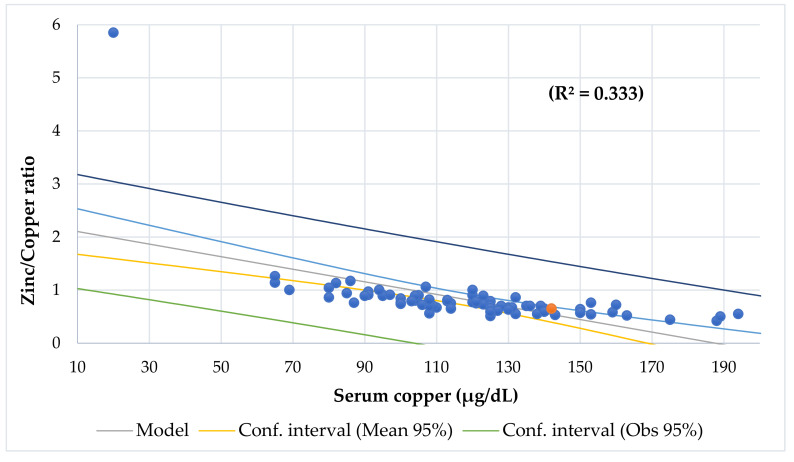
Regression Zinc/Copper ratio by serum copper (µg/dL).

Table 3 shows the differences between participants with chronic diseases, Table 4 shows the Odds Ratio in the entire series, and Table 5 shows the association between BCS via BIA with anthropometric assessment. Serum Cu and Zn levels had a direct association when adjusted by age (Figure 2). Serum Cu (*r* = −0.386, *p* = 0.000) and Cu/Zn ratio (*r* = −0.380, *p* = 0.001) had an inverse and significant correlation with age. Lineal regression analysis showed that serum Cu decreased significantly with age, but there was no variation for serum Zn (Figure 3). Mean serum Cu and the Cu/Zn ratios were significantly higher in children (128 μg/dL, 1.49) than in adolescents (106 μg/dL, *p* = 0.001; 1.25, *p* = 0.004). The risk of finding altered Cu levels was higher in male, children, children under 5 years (eight cases), under 5 age-for-50° Height (10 cases), and in patients with high ESR than in females, adolescents, children >5 years, children >5 age-for-50° Height, and patients with normal ESR. Although mean serum Cu in males (123 μg/dL) was higher than in females (113 μg/dL), this difference was not significant. The probability of finding abnormal serum Cu (OR 3.9) and hypercupremia (OR 5.3) cases in males was higher than in females. A significant positive association was found between serum Cu and Cu/Zn ratio (*p* < 0.001) and a negative association with Zn/Cu ratio (*p* < 0.001) (Figure 4 and Figure 5). Table 6 and Table 7 show the meaningful multiple regression analysis between serum Cu and Cu/Zn and Zn/Cu ratios with other nutritional parameters studied throughout the series and according to nutritional status, respectively.

**Figure 6:**
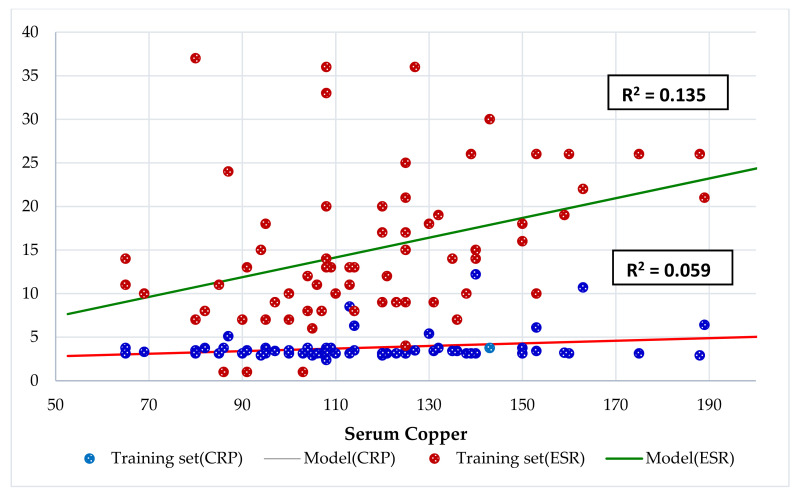
Regression C-reactive protein (CRP) and erythrocyte sedimentation rate (ESR) by serum Cu (µg/dL).

CRP and ESR levels were normal except in eight (11%) and 19 patients (24%), respectively. Twenty-four percent of patients had high ESR and 77% of them had hypercupremia. Although CRP and ESR did not have a significant correlation to each other (*p* > 0.05), ESR had a direct and significant association with Cu (*r* = 0.324 **, *p* = 0.006) and Cu/Zn ratio (*r* = 0.468 **, *p* = 0.000) compared to CRP, which only had a significantly lower correlation with Cu (*r* = 0.239 *, *p* = 0.044) but not with Cu/Zn ratio (*r* = 0.228, *p* = 0.056). Linear Regression analysis (Figure 6) showed that serum Cu had a significant association with ESR (R^2^ = 0.135, *p* = 0.002) and CRP (R^2^ = 0.059, *p* = 0.0047). Participants with elevated CRP had significantly higher mean serum Cu (136 μg/dL) and Cu/Zn ratio (1.61) and a meaningfully lower Zn/Cu ratio (0.64) than patients with normal CRP (115 μg/dL, *p* = 0.036; 1.35, *p* = 0.047; 0.79, *p* = 0.029). Patients with elevated ESR had significantly higher mean serum Cu (136 μg/dL) and Cu/Zn ratio (1.69) and a meaningfully lower serum Zn (80 μg/dL) than patients with normal ESR (110 μg/dL, *p* = 0.001; 1.25, *p* = 0.000; 89 μg/dL, *p* = 0.011). Ten (77%) and three (23%) patients out of 13 with hypercupremia had elevated ESR and CRP, respectively. Hypercupremic patients had normal ERS and CRP (Table 2). The probability of finding an elevated ESR (OR 11) in patients with hypercupremia and in patients with abnormal serum Cu (OR 5) was higher than those with normal Cu levels. 

Serum Cu and Cu/Zn and Zn/Cu ratios had a significant association with IGF-1 in the entire series and in the undernutrition group. Means of IGF-1 and IGFBP3 in the undernutrition group were significantly lower than means in the eutrophic and obesity groups. In the eutrophic group, IGF-1 was associated with serum Cu, IGF-1, and serum P with Cu/Zn ratio, and IGF-1 and IGFBP-3 with a Zn/Cu ratio. In the obesity group, serum P was associated with Cu/Zn and Zn/Cu ratios, and IGF-1 with serum P was associated with serum Cu. In the entire series, serum Ca and P were normal and serum P had a positive and significant correlation with serum Ca (*r* = 0.455 **, *p* = 0.000) and Cu (*r* = 0.285 *, *p* = 0.013). Furthermore, IGF-1 had a negative association with serum Ca (*r* = −0.231 * *p* = 0.046) and P (*r* = −0.287 *, *p* = 0.012). Multiple regression analysis showed a significant association between serum P with serum Ca, Zn, and IGF-1 (R^2^ = 0.337, *p* = 0.000). 

In the obesity group, the serum Cu and Cu/Zn ratio were associated with the height-for-age and the height-for-age Z score. In the undernutrition and eutrophic groups, serum Cu and Cu/Zn and Zn/Cu ratios were associated with kg mass muscular, suprailiac skinfold Z-score, waist/hip and waist/height ratio, hip and wrist perimeters, arm muscular area, height-for-age, and BMI Z-score, in different relationships. There were 46% (6/13 cases) and 23% (2/13 cases) of patients with hypercupremia and weight-for-age and height-for-age < 2SDS, respectively. Twenty-two wasting patients (28%) had higher serum Cu (120 µg/dL), Zn/Cu ratios (0.9), and normal Cu/Zn ratios than normal-weight patients (116 µg/dL, *p* = 0.012; 0.7, *p* = 0.011). Thirteen patients with stunted growth (17%) had higher Zn/Cu ratios (1.1) and normal serum Cu and Cu/Zn ratios than patients with normal height (0.7, *p* = 0.000). Multiple regression analysis showed that serum Cu was associated with height-for-age and height-for-age Z-score and triceps skinfold, and the Cu/Zn and Zn/Cu ratios were associated with height-for-age and triceps skinfold. The probability of finding a low weight-for-age in patients with abnormal serum Cu (OR 4) was higher than those with normal weight.

Mean BCS of 1924 ± 88 was normal. There was no significant difference in serum Cu and Cu/Zn and Zn/Cu ratios between patients with low and normal BCS. In the entire series, only five cases had low BCS and only two cases with hypercupremia had a risk of osteoporosis. BCS had a negative and significant correlation with serum Cu, the Cu/Zn ratio, and a positive association with the Zn/Cu ratio. In the whole series, multiple regression analysis showed that BCS was associated with age, BMI, kg muscle mass by anthropometry, and muscle mass by BIA (R^2^ = 0.663; *p* = 0.000). In the obesity group, BSC was associated with age and BMI (R^2^ = 0.699; *p* = 0.000), in the undernutrition group with age-for-50° height (R^2^ = 0.814 **; *p* = 0.000), and in the eutrophic group with Kg muscle mass by anthropometry (R^2^ = 0.791; *p* = 0.000). Multiple regression analysis showed that BCS in the entire series was associated with serum Cu and Cu/Zn and Zn/Cu ratios, and, by nutritional groups, only in the group of obese and eutrophic patients. Serum Cu and Cu/Zn and Zn/Cu ratios had different significant associations. In the undernutrition group, the BMD measured through the BSC was significantly associated with age, weight, height, weight for height, muscle mass, and fat by anthropometry (Table 5). This was not the case with BMI and fat mass and muscular through the BIA.

As for the dietary survey intake, the daily intake for the entire series was hyperproteic (276% DRI), with high consumption of cholesterol (266% DRI), slightly low intake of carb (79.5% DRI), and a normal total lipid intake (111% DRI). In the whole series, multiple regression analysis showed that serum Cu had a meaningful association with the intake of fiber, magnesium, and vitamin B6, the Cu/Zn ratio had a meaningful association with fiber consumption, and the Zn/Cu ratio had a meaningful association with the intake of vitamins B1, B2, and B6. Furthermore, the probability of finding deficient folic acid intake in patients with abnormal serum Cu (OR 8) and high vitamin A intake in patients with hypercupremia (OR 4) was higher than those with normal intakes of folic acid and vitamin A. In our study, fiber was the only one that had an inverse and significant correlation with serum Cu and Cu/Zn ratios. 

In the entire series, multiple regression analysis showed that serum Cu was associated with prealbumin, gamma-glutamyl transferase (GGT), and cardiovascular risk, the Cu/Zn ratio was associated with prealbumin and total bilirubin, and the Zn/Cu ratio was associated with GGT, alanine aminotransferase (ALT), prealbumin, and total bilirubin. There were 31% and 15% of hypocupremic patients with low prealbumin and high cholesterol levels, respectively. Prealbumin and GGT were the only ones that had a significant positive correlation with the Zn/Cu ratio and a negative association with serum Cu and the Cu/Zn ratio. In addition, multiple regression analysis throughout the series showed that serum Cu was associated with leucocytes, hemoglobin, the mean corpuscular hemoglobin concentration (MCHC) and neutrophils, IgG1, IgG4, and CD4 T-lymphocytes, while the Cu/Zn ratio was associated with hemoglobin and neutrophils, IgG3, and IgG4, and the Zn/Cu ratio was associated with mean corpuscular volume (MCV), ESR, CRP, and IgG4. Twenty-nine percent (5/17 cases) of patients with hypercupremia had iron deficiency anemia (IDA). The probability of finding leukocytosis (OR 8) in patients with abnormal serum Cu was higher than those with normal Cu levels.

## 4. Discussion

It is interesting discovering that not much is known about copper metabolism in chronically ill children and adolescents. To the best of our knowledge, this is the first study to explore serum Cu levels and the Cu/Zn ratio and its association with nutritional indicators in a series of children and adolescents with chronic diseases. In the entire series and according to nutritional status, the mean serum Cu and Zn and the Zn/Cu ratio were normal, but the Cu/Zn ratio was high. Serum Cu decreased significantly with age and was meaningfully higher in children than adolescents. The Cu/Zn ratio was also meaningfully higher in children than adolescents. The risk of finding altered Cu levels was higher in children and males than in adolescents and females. Sixty-four percent of the patients had marginal zinc deficiency. Although all hypozincemic and hypocupremic patients had dietary Zn deficiency, only 65% of hypercupremic patients had deficient Zn intake. There were 87% of patients with a Cu/Zn ratio > 1.0 and 5% with a Cu/Zn ratio > 2.0. Only one case had a Zn/Cu ratio > 4.0. Multiple regression analysis showed that serum copper and Cu/Zn and Zn/Cu ratios had significant associations with nutritional parameters studied in the whole series and according to nutritional groups. 

### 4.1. Serum Cu Levels

Up to now, pediatric reference intervals for serum Cu have often been difficult to set up [43]. In our series of patients with chronic disease aged from 1 to 19, the mean serum Cu (118 μg/dL) was normal and there was no significant difference with the mean serum copper in a series of CF patients (113 μg/dL, *p* = 0.001) [27]. However, our mean serum Cu was significantly lower compared to the mean serum Cu of the German study carried out in children aged between 1 month to 18 years (20.4 ± 4.9 μmol/L or 129.6 μg/dL, *p* = 0.001) [44], and the study conducted in 120 healthy children (1 to 18 years of age) (134.5 μg/dL, *p* = 0.000) who did not receive any vitamins and mineral supplements [7] as in our series. This may mean that in chronically ill children and adolescents, serum copper levels could be lower than in healthy children.

Interestingly, serum Cu changes in relation to age and gender [45]. Results showed that serum Cu and the Cu/Zn ratio had an inverse and significant correlation with age. Serum copper and zinc levels had a direct association when they were adjusted by age (Figure 2), and linear regression analysis showed that serum Cu decreased significantly with age (Figure 3), as observed in a previous study conducted in a series of CF patients [27]. Furthermore, the mean serum copper and the Cu/Zn ratios were significantly higher in children than adolescents, and the probability of finding altered Cu levels and hypercupremic cases in children (OR 3.6 and 14), children under 5 years (OR 5.8 and 7.3), and under 5 age-for-50° Height (OR 4.4 and 6.9) was higher than in adolescents, children >5 years, and children >5 age-for-50° Height, respectively. Similarly, in preschool children with attention deficit/hyperactivity disorder (ADHD), age was associated with a significant increase in Cu and Cu/Zn values [46]. As in our series, Acosta et al. (2010) reported that Cu levels had an inverse and significant correlation with age, and children under the age of 10 years had higher Cu levels than children over 10 [47]. In healthy Greek children, significantly higher Cu levels were found in children under the age of 5 than in children aged between 6 to 10 and in children >10 years old [48], as proven in our series. However, in some studies, a decrease in serum Cu has been reported as age increases [49]. In adolescents, this may be due to a change between extra and intracellular Cu storage [44]. These results are important because they could reveal that chronically ill children under 10 years of age, especially those under 5, could develop an altered serum copper status. 

Regarding gender, some studies found no statistically significant differences in serum Cu in healthy children [50], but other studies revealed that women had significantly higher serum Cu than men [27,51,52]. In 560 Kuwaitis (15–80 years), Cu in females (158 µg/dL) was significantly higher than in males (133 µg/dL, *p* < 0.0001) [53]. Furthermore, Malavolta et al. (2010) found that in elderly patients (>70 years) the Cu/Zn ratio was higher in women than men and increased with age [20]. However, the data obtained in our study of children and adolescents with chronic diseases showed that the mean serum Cu in males (123 μg/dL) was higher than in females (113 μg/dL). Although this difference was not significant, the probability of finding abnormal serum Cu (OR 3.9) and hypercupremia (OR 5.3) cases in males was higher than in females. These findings provide evidence that suggests that, in children and adolescents with chronic diseases, males could develop an altered copper status.

Both copper deficiency and excess copper have been recognized as potential, major health problems for infants and children worldwide. The percentage of patients with hypercupremia and Cu deficiency changes depend on different studies [54]. In this study, 22% of the participants had abnormal mean serum Cu, 13 cases had hypercupremia, and four cases had hypocupremia. A 2-year-old boy with mitochondrial neuropathy and severe neurological involvement had a very low serum Cu level (20 μg/dL) and a normal serum Zn (117 μg/dL). His neurological status made it impossible to assess whether he might have symptoms due to Cu deficiency and his diet was improved. We must bear in mind that Duncan et al. reported that low plasma copper (6 mol/L or 38 μg/dL), together with high plasma zinc (18 mol/L or 117.7 μg/dL), is an important predictive factor for the diagnosis of zinc-induced Cu deficiency (ZICD) [55]. Although another 15-year-old male with the severe malabsorptive syndrome had a posterior cord peripheral neuropathy, his copper and zinc levels of 65 μg/dL and 82 μg/dL, respectively, were not compatible with a diagnosis of ZICD. In our series, the prevalence of hypocupremia (5%) and hypozincemia (6%) was higher than the values reported by Abiaka et al. They reported 0.36% and 0.53% of Cu and Zn deficiency in the Arab population (15–80 years of age) [53]. Results showed that 10 patients had serum Cu < 0.90 mg/L and one patient < 0.45 mg/L. According to Cordano, the serum Cu concentrations < 0.90 mg/L [54] and particularly < 0.45 mg/L strongly support the diagnosis of Cu deficiency [56]. Uauy et al. reported that Cu deficiency is more commonly an acquired condition induced by the imbalance between need and dietary Cu supply [57]. Therefore, our series presented a moderate percentage of patients with altered copper levels.

This study also found that serum Cu and Cu/Zn, and Zn/Cu ratios had a significant association with insulin-like growth factor-1 (IGF-1) in the entire series and in the undernutrition group. In the eutrophic group, IGF-1 was associated with serum Cu, IGF-1, and P with Cu/Zn ratio, and IGF-1 and IGFBP-3 with Zn/Cu ratio. Under normal circumstances, IGFBP-3 is the main carrier of circulating IGF-1, due to its high affinity. In an in vitro study, it was observed that copper supplementation in a culture medium containing 15% fetal calf serum (FCS) could promote the autocrine secretion of IGF-1 and IGFBP-3 and stimulate chondrocyte proliferation [58]. Furthermore, an animal study, which was carried out in 60 weanling pigs, concluded that the effects of Cu in their diet to supplement their growth was related to the increased serum levels induced by Cu. High dietary Cu increases the concentrations of serum growth-related hormones, growth hormone (GH), insulin (INS), IGF-1, and IGFBP-3, which improves growth performance [59].

Phosphorus is another important element in the body and, together with serum Ca, it influences bone health [60]. In the entire series, serum P was normal and had a positive and significant correlation with serum Ca (*r* = 0.455 **, *p* = 0.000) and Cu (*r* = 0.285 *, *p* = 0.013). Furthermore, IGF-1 had a negative association with serum Ca (*r* = −0.231 * *p* = 0.046) and P (*r* = −0.287 *, *p* = 0.012). Multiple regression analysis showed a significant association between serum P with serum Ca, Zn, and IGF-1 (*R^2^* = 0.337, *p* = 0.000). In a study of 747 children of short stature, there was a positive correlation between serum P and IGF-1 SDS when the serum P concentration was greater than 3.9 mg/dL [61]. In the obesity group, serum P was associated with the Cu/Zn and Zn/Cu ratios, and IGF-1 with serum P was associated with serum Cu. Copper can promote Ca and phosphorus deposits and collagen synthesis [52]. These findings are particularly significant because they could indicate that, in children and adolescents with chronic diseases, there is a relevant association between Cu, P, IGF-1, and IGFBP3, which should be studied. 

### 4.2. Phenotypical Characteristics 

Research has found that anthropometric and body compositions have different relationships with serum Cu and Cu/Zn and Zn/Cu ratios (Table 6 and Table 7). Multiple regression analysis showed that serum Cu was associated with height-for-age and height-for-age Z-score and triceps skinfold, and the Cu/Zn and Zn/Cu ratios were associated with height-for-age and triceps skinfold. According to Barrientos et al., different skinfolds, fat mass, muscle mass, and bone mass correlated positively and negatively with trace elements such as copper. In addition, in a series of athletes, Cu had a negative correlation with the subscapular skinfold, fat mass, muscle mass, and bone mass [62]. Interestingly, 46% (6/13 cases) and 23% (2/13 cases) of the patients with hypercupremia had weight-for-age and height-for-age < 2SDS, respectively. Twenty-two wasting patients (28%) had higher serum Cu (120 µg/dL) and Zn/Cu ratios (0.9) and normal Cu/Zn ratio than normal weight patients (116 µg/dL, *p* = 0.012; 0.7, *p* = 0.011). Thirteen patients with stunted growth (17%) had higher Zn/Cu ratios (1.1) and normal serum Cu and Cu/Zn ratios than patients of normal stature (0.7, *p* = 0.000). The probability of finding low weight-for-age in patients with abnormal serum Cu (OR 4) was higher than those of normal weight.

These results are not surprising because Castro et al. (2017) reported a strong association between serum Cu levels with height or weight [63], and Laitinen et al., in a series of 3415 Finnish children and adolescents, concluded that Cu levels were correlated with stature between the ages of 3 to 18 [64]. What is more, a study conducted on 100 school children between 10 and 14 years of age showed that there was a significant difference among different classifications of height-for-age with respect to the Cu/Zn ratio. This ratio was significantly higher in schoolchildren with mild wasting compared to normal children [65]. Girls who were in the 5th percentile or greater for height were found to have higher serum Cu levels than girls in other height categories [66]. Sorokman et al. (2020), in a series of 42 children aged 3 to 15 with different types of short stature, found lower plasma Cu levels than in the control group [67]. These results are important because they point out groups of patients with Cu deficiency and height that had not been identified [64].

It is necessary to highlight that, in a series of CF patients, there was a direct association between serum Cu levels and BMI [27]. Similarly, Abiaka et al. reported that Cu levels were positively associated with BMI values (*r* = 0.302, *p* < 0.001) in a group of Arabs [53]. In a study, the Cu level in the groups of overweight (15 cases) and obese (30 cases) men was significantly higher than in the control group (*p* = 0.006) [68]. In contrast, in our series, although the mean serum Cu in the undernutrition group (114 μg/dL) was lower than in the eutrophic (122 μg/dL) and obese patients (119 μg/dL), this difference was not significant. Eight out of 21 malnourished children and one out of four eutrophic patients had lower weight-for-age and abnormal serum Cu. According to researchers, the risk of hypocupremia was higher in malnourished patients [11,57,69]. This fact is interesting because it has been shown in a meta-analysis that a higher serum Cu level could be associated with the risk of obesity in children and adults [70]. 

### 4.3. Bone Densitometry

With regard to bone densitometry by BCS, the mean of 1924 ± 88 was normal. There was no significant difference in serum Cu and Cu/Zn and Zn/Cu ratios between patients with low and normal BCS. In the entire series, only five cases had low BCS and only two cases with hypercupremia had a risk of osteoporosis. BCS had a negative and significant correlation with serum Cu (*r* = −0.521 **; *p* = 0.000) and the Cu/Zn ratio (*r* = −0.484 **; *p* = 0.000) and a positive association with the Zn/Cu (*r* = 0.447 **; *p* = 0.000) ratio. Linear regression analysis showed that BCS had a positive correlation with serum Cu in a series of patients with CF [27]. In the whole series, multiple regression analysis showed that BCS was associated with age, BMI, kg muscle mass by anthropometry, and muscle mass by BIA (R^2^ = 0.663; *p* = 0.000). Weight and body composition are important modifiable determinants of bone mass. Multiple studies have shown that BMD is strongly associated with lean body mass, which is directly correlated with BMI, and that increased adiposity could be associated with an increased risk of fractures [71]. 

In the obesity group, BSC was associated with age and BMI (R^2^ = 0.699; *p* = 0.000); in the undernutrition group BSC was associated with age-for-50° height (R^2^ = 0.814 **; *p* = 0.000); and in the eutrophic group BSC was associated with Kg muscle mass by anthropometry (R^2^ = 0.791; *p* = 0.000). Multiple regression analysis showed that BCS in the entire series was associated with serum Cu and the Cu/Zn and Zn/Cu ratios, and by nutritional groups, only in the group of obese and eutrophic patients. In 83 children with chronic pancreatitis (CP), BMI had a significant correlation with bone mineral density (BMD), and 41% of these patients with mainly mild undernutrition had a lower percentage of body fat and BMD [72]. In the undernutrition group, the BMD measured through the BSC was significantly associated with age, weight, height, weight for height, muscle mass, and fat by anthropometry (Table 5), but not so with BMI and fat mass and muscular through the BIA. This fact is interesting and perhaps answers why BSC in this specific group was not associated with serum Cu or with the Cu/Zn and Zn/Cu ratios.

We need to consider that Cu has a positive effect on osteoblast proliferation and function and indirectly promotes osteogenic and adipogenic differentiation of bone marrow mesenchymal stem cells (BMSCs) [73], playing a key role in the crosslinking of collagen and elastin [74]. Collagen is a major component of the extracellular matrix of bone tissue and participates with elastin in the production of the bone matrix [75]. Cu deficiency bone abnormalities include osteoporosis (during periods of rapid growth), long bone and rib fractures, epiphyseal separation, fraying and cupping of metaphysis with spur formation, and subperiosteal new bone formation [76]. In a review on the correlation of blood Cu, daily Cu intake, and Cu supplementation with BMD, only one study showed differences in Cu levels between osteoporotic and healthy women, although only in the case of women 45–59 years of age [77]. Additionally, in 8224 American adults of the National Health and Nutritional Examination Surveys (NHANES 2007–2018), total Cu intake was positively associated with increasing BMD and negatively associated with the risk of osteoporosis [78]. 

### 4.4. Dietary Intake Survey

As far as diet is concerned, serum Cu and the Cu/Zn and Zn/Cu ratio had different significant associations with the nutrients analyzed from the reported daily intake (Table 1, Table 6, and Table 7). Furthermore, the probability of finding deficient folic acid intake in patients with abnormal serum Cu (OR 8) and high vitamin A intake in patients with hypercupremia (OR 4) was higher than those with normal intakes of folic acid and vitamin A. Gonoodi et al. reported that serum Cu levels were inversely related to dietary energy and fat intake [66,79]. However, in our study, fiber was the only one that had an inverse and significant correlation with serum Cu and Cu/Zn ratios. According to Kaslow, although the consumption of Cu may be below the recommended level, its deficiency is relatively rare [40]. Several dietary factors can have adverse effects on the bioavailability of ingested copper, including carbohydrates, iron, zinc, certain amino acids and proteins, molybdenum, and vitamin C [80]. Moreover, it was found that 64% had deficient zinc intake and 64% had a high risk of marginal zinc deficiency (Table 1 and Table 2). Although all patients with hypozincemia (five cases) and hypocupremia (four cases) had dietary Zn deficiency, only 65% of the patients with hypercupremia (13 cases) had a deficient Zn intake. The dietary intakes of Cu and Zn decrease with age and, thus, both are potentially deficient nutrients in aging [81]. High dietary Zn intake (more than 50 mg/day day-to-day) for extended periods impairs intestinal Cu absorption. This is explained by induction of metallothioneins (MT), a Cu-binding protein, in intestinal epithelial cells [82]. High levels of MT due to increased Zn can cause reduced absorption of copper. Instead, it was found that high doses of Cu affect the Zn nutritional status [83]. ZICD can result in erythropoietin-resistant anemia [84]. Cu deficiency myelopathy (CDM) has only been described in the last decade and represents a treatable cause of non-compressive myelopathy that closely mimics subacute combined degeneration due to vitamin B12 deficiency [34].

### 4.5. Biochemical Analysis

Biomarkers for the identification of Cu status are still being defined [85]. In the entire series, multiple regression analysis showed that serum Cu was associated with prealbumin, GGT, and cardiovascular risk, the Cu/Zn ratio was associated with prealbumin and total bilirubin, and the Zn/Cu ratio was associated with GGT, ALT, prealbumin, and total bilirubin. Although the results yield statistically significant differences between serum Cu and Cu/Zn and Zn/Cu ratios and biochemical indicators by nutritional status (Table 6 and Table 7), some of them are backed up by other studies. In 100 SARS-CoV2-positive pregnant women, in the first and third trimesters, serum Cu was associated with ALT, and, in the second trimester, with blood urea nitrogen (BUN) and creatinine [86], as in our series. In our study, prealbumin and GGT were the only ones that had a significant positive correlation with Zn/Cu ratio and a negative association with serum Cu and Cu/Zn ratios. In contrast, in a series of CF patients, GGT had a significant positive correlation with the Cu/Zn ratio and a negative association with the Zn/Cu ratio [27]. GGT is an early and sensitive basic parameter for estimating oxidative stress and inflammation. In 281 adults, Peng et al. found a significantly positive correlation between GGT and Cu, indicating that GGT may be a biomarker to evaluate serum Cu in an adult population [87]. In our study, 31% (4/13 cases) of hypercupremic patients had low prealbumin. Prealbumin is a sensitive indicator of malnutrition and inflammation, and it has been associated with mortality in the elderly. In 185 free-living elderly women with low-grade subclinical inflammation, elevated Cu was associated with a decrease in serum prealbumin [88]. In our series, 15% (2/13 cases) of patients had high cholesterol levels. Cholesterol is one of the CVD’s risk factors. In 1427 children and adolescents from a nationally representative sample of the NHANES from 2011–2014, serum Cu was strongly associated with total cholesterol [89]. Alanine aminotransferase is a specific liver test. In 175 patients with hepatocellular carcinoma (HCC), serum Cu was positively correlated with ALT [90]. 

### 4.6. Blood Analysis and Inflammatory Response

Results showed that there was significant association between serum Cu and Cu/Zn and Zn/Cu ratios with blood analysis and immune response. Anemia is a clinical sign of both iron and Cu deficiency [91]. In our study, there were 29% (5/17 cases) with hypercupremia and IDA. In 60 Turkish children aged between 1 to 14 with IDA, there were statistically significant negative correlations between hematological parameters and serum Cu levels [92]. The utilization of iron in bone marrow requires Cu since Cu deficiency affects hemoglobin production despite normal serum iron levels [93]. Furthermore, in this study, the probability of finding leukocytosis (OR 8) in patients with abnormal serum Cu was higher than those with normal Cu levels. Copper plays a crucial role in the development, maturation, and proper functioning of the immune system [94]. The maturing immune system relies on Cu and Zn [95], especially for antibody production (both Cu and Zn), function of neutrophils and monocytes (Cu) [96], and the viability, proliferation, and differentiation of cells of both the innate and adaptive immune system (Zn), as well as for the maintenance of skin and mucosal barriers (Zn) [95,96]. Higher Cu levels can significantly decrease the number of circulating neutrophils, antibody titer, CD4/CD8 ratio, and NK cell activity [97].

CRP and ESR levels were normal except in eight (11%) and 19 patients (24%), respectively. Although CRP and ESR did not have a significant correlation to each other (*p* > 0.05), ESR had a direct and significant association with Cu (*r* = 0.324 **, *p* = 0.006) and Cu/Zn ratio (*r* = 0.468 **, *p* = 0.000) compared to CRP, which only had a significant lower correlation with Cu (*r* = 0.239 *, *p* = 0.044) but not with Cu/Zn ratio (*r* = 0.228, *p* = 0.056). Linear Regression analysis (Figure 6) showed that serum Cu had a significant association with ESR and CRP. Twenty-four percent of the patients (19/78 cases) had high ESR and 77% of them had hypercupremia. These high ESR subjects had a meaningfully higher Cu/Zn ratio and lower serum Zn than normal ESR patients. Subjects with elevated CRP had a significantly high mean serum Cu and lower Zn/Cu ratio than patients with normal CRP. In contrast, all hypocupremic patients had normal ERS and CRP. We must consider that the probability of finding an elevated ESR (OR 11) in patients with hypercupremia and in patients with abnormal serum Cu (OR 5) was higher than those with normal Cu levels. Dizdar et al. found that Cu levels were significantly higher in patients with soft tissue infection than in normal subjects, and they also found a positive correlation between serum Cu levels and ESR [39], as in our study. Bui et al. found that CRP was associated with serum ferritin and Cu concentrations in apparently healthy school children [54]. Schneider showed that, in patients with Crohn disease (CD) and ulcerative colitis (UC), CRP was positively correlated with serum Cu and the Cu/Zn ratio in both CD and UC [98]. Our results indicated that, in this series of children and adolescents with chronic disease, the serum Cu and Cu/Zn ratio showed a stronger and more significant association with ESR than with CRP. 

### 4.7. Copper-to-Zinc Ratio

The results of this study suggest significant associations between the Cu/Zn ratio and adverse clinical outcomes. A significant positive association was found between the serum Cu and Cu/Zn ratios (*p* < 0.001) and a negative association with the Zn/Cu ratio (*p* < 0.001) (Figure 4 and Figure 5). Eighty-seven percent of patients (68 cases) had a Cu/Zn ratio > 1.0, which means there was a pattern of high Cu and low zinc, which is characteristic of an inflammatory condition [99]. Additionally, the Cu/Zn ratio of ≥1.1 can be an effective marker for the diagnosis of taste disorders derived from Zn deficiency [100]. This 87% of patients with a high Cu/Zn ratio contrasted with a study carried out in 68 children with ADHD, in which the serum Cu/Zn values were 11% higher than those in their control group [46]. Although all children with hypocupremia had a normal Cu/Zn ratio, our results showed that all patients with hypozincemia and hypercupremia had a higher Cu/Zn ratio > 1.00.

Additionally, in our series, four patients with hypercupremia (23%) had Cu/Zn ratio > 2.0. Only one 3-year-old eutrophic boy (8%) had hypercupremia, hypozincemia, dietary Zn deficiency, high ERS, and a Cu/Zn ratio > 2.0, and another undernutrition 2-year-old boy (25%) had deficient Zn intake, hypocupremia, and high Zn/Cu ratio. If this ratio exceeds 2, it will indicate severity of bacterial infection [101]. Bogden reported elevated Cu levels in tuberculous patients with reduced Zn levels and a Cu/Zn ratio > 2.0 in 87% of cases [102]. Furthermore, an increase above 2.0 in the elderly usually reflects an inflammatory response or a decreased nutritional Zn status [20]. Acute infections alter Cu and Zn metabolism, while deficiencies increase the risk of infection [103]. Unlike zinc, Cu concentration increases during the acute-phase response [104] and in patients with soft-tissue infections [39]. To be exact, conditions associated with increased inflammatory and/or deficient nutrition may be signaled by decreased Zn [105] and/or increased Cu [20], leading to an altered Cu/Zn ratio [106]. Therefore, in patients with an active disease, Cu levels could be higher than in patients in remission [107]. According to Lee et al., this association could be explained by the importance of Cu in collagen tissue repair [108]. 

Unexpectedly, this Cu/Zn ratio was shown to be a better predictor of disease severity and/or mortality than Cu levels [20]. This ratio may be a useful tool as a prognostic and predictive factor for a multitude of pathological and pre-pathological conditions and comparable to other inflammatory biomarkers, such as CRP and ESR [20,106] or, for instance, in patients with HIV [109]. The diagnostic value of the Cu/Zn ratio as a disease marker was also shown in other diseases, such as Sickle cell disease (SCD) [110], autism, attention-deficit hyperactivity disorder, hypertension [37], and other degenerative diseases. The Cu/Zn ratio also could be used as a clinical indicator for diagnosis of digestive cancers [111], hepatocellular cancers [90], bladder cancer [112,113], ovarian cancers [113], and lymphoproliferative disorders [114]. It has been seen in patients with an increased risk of breast cancer [115] and patients with metastasis [116]. It is a useful early prognostic biomarker of early-onset infection in term and preterm neonates [17]. Moreover, it has been associated with the risk of CVD death, malignancy, and all-cause mortality in the very elderly [106]. The combination of low plasma Cu and high plasma Zn is strongly predictive for the diagnosis of ZICD [55]. 

Our research found that Cu/Zn ratio is associated with different inflammatory markers as well as nutritional indicators such as prealbumin. The Cu/Zn ratio appears to have an important impact on metabolism, indicating that these trace elements play an important role in the pathogenesis of metabolic disease [117]. These findings agree with the results of Guo et al., who found significant negative correlations of the Cu/Zn ratio with nutrition-related parameters (BMI, creatinine, hemoglobin, and albumin) and antioxidant (vitamins C and E) levels [23]. As we have shown, previous studies revealed the validity of the Cu/Zn ratio for the severity of nutritional status, inflammation, oxidative stress, immune dysfunction, and infection associated with Zn deficiency [20,23,38]. Therefore, the Cu/Zn ratio appears also to reflect the severity of Zn deficiency [100]. 

Multiple regression analysis showed, in our study, an important association between Zn/Cu ratio and other nutritional indicators. Plasma Zn/Cu ratio may be a biomarker that indicates stress on the MT system of children with autism spectrum disorder (ASD). Children with ASDs appear at risk of Zn deficiency (<66 µg/dL) and Cu toxicity (>153 µg/dL), showing a low Zn/Cu ratio (72.6% < 0.7) and decreased function of the MT system, which contributes to dysregulated neurotransmitter system functioning, decreased zinc finger protein activity, and diminished zinc-dependent gastrointestinal enzymatic activity [118,119]. According to this parameter (Table 2), 41% of the patients in our series had a Zn/Cu ratio < 0.7, indicating stress on the MT system [38,119]. As mentioned by Van et al. (2020), considering the antagonistic effect of Zn and Cu, when the ratio of Zn to Cu in serum is close to 1:1, the immune response to infectious agents is more effective [120,121]. Because Zn inhibits intestinal Cu absorption, the risk of Cu deficiency increases when the Zn/Cu molar ratio is high [122]. A high serum Zn/Cu ratio is also found in people with debilitating conditions, such as elderly, hospitalized patients [123]. In patients with lung cancer, the risk of mortality was almost doubled in patients with the lowest values of blood Zn/Cu ratio compared with those with the highest values [116]. We recognize that the Zn/Cu relationship is the inverse of Cu/Zn [38], as observed in our study (*p* < 0.001), and we must obtain significant and inverse associations with the different nutritional indicators studied, as occurred in a study carried out in patients with CF [27]. However, this did not happen in our study, where we found differences between Cu/Zn and Zn/Cu with the nutritional indicators studied (Table 5 and Table 6). We believe that these differences should be considered and studied.

At this point, we need to consider some highlights. Our results suggest that, in chronically ill children and adolescents, the serum level of Cu could be lower. Serum Cu and the Cu/Zn ratio were inversely related to age. Children under 10 years of age and especially those under 5 years of age could develop altered levels of serum Cu (hypercupremia). Both children and males are more likely to develop altered levels of copper compared to adolescents and females. The relevant association between Cu, P, IGF-1, and IGFBP3 should be studied. Height-for-age could be a bioindicator of Cu status. Malnourished children are at risk for Cu deficiency, and this could be reflected in their BMD. There is a high risk of marginal zinc deficiency in patients with hypercupremia. Liver and malnutrition markers are related to Cu status markers. A strong and significant association was observed between ESR first with the Cu/Zn ratio and second with serum Cu, which would indicate that the Cu/Zn ratio may be used as an inflammatory marker. Finally, this study demonstrated that the serum Cu level had a meaningful association with anthropometric, biochemical, dietary, body densitometry, and body composition indicators. Considering all the highlights, we could indicate that the Cu/Zn ratio reflects the inflammatory status and a high risk of Zn deficiency in our series of children and adolescents with chronic disease.

We need to bear in mind that Cu and Zn can be determined in biological samples by various methods, including AAS (Atomic Absorption Spectroscopy), GF-AAS (Graphite Furnace -AAS) or Electrothermal Atomic Absorption Spectrometry (ETAAS), Inductively Coupled Plasma-Optical Emission Spectroscopy (ICP-OES), ICP-Mass Spectroscopy (ICP-MS), etc. Depending on the number of samples and the availability of equipment, the results of all of them are perfectly comparable if due precautions are taken in their analysis [124]. Furthermore, methods like AAS are still robust, highly sensitive, and low-cost methods for the measurement of single elements and can be used as reference methods [125]. Therefore, our findings may have important implications for the assessment of Cu and Zn status. As demonstrated in this study, serum Cu and Zn levels in chronically ill children can be affected. The results respond to the main objective of this study and justify continuing with the investigation into the relationship between the nutritional status of children and adolescents with chronic disease and Cu status. A limitation of this study is the small number of participants per group of nutritional status. Its strengths lie in the determination of serum Cu levels and the Cu/Zn and Zn/Cu ratios and its relationship with different anthropometric, biochemical, dietary, bone densitometry, and body composition indicators. The issue of background knowledge should be investigated further. As a result, the authors suggested the implementation of multicenter trials to improve the understanding of Cu status in these patients and to determine the necessary and appropriate amount of Cu supplementation to improve the nutritional status of patients with chronic disease when necessary. These findings should be verified in larger groups of children so as not to miss an easily avoidable risk factor for poor development. Therefore, there is a critical need to standardize serum Cu and Zn levels to assess specific needs not only for healthy children and adolescents but also for children and adolescents with chronic diseases.

## 5. Conclusions

Serum copper and Cu/Zn and Zn/Cu ratios are important bioindicators of nutritional status in children and adolescents with chronic diseases and are related to other significant nutritional indicators. Serum Cu and Cu/Zn ratio were inversely related to age. Children and males had more risk to develop abnormal serum copper and hypercupremia than adolescents and females, respectively. Serum copper may be used as a biomarker of inflammation like erythrocyte sedimentation rate. The Cu/Zn ratio may indicate an inflammatory status and high risk of zinc deficiency in children and adolescent with chronic diseases. 

## Figures and Tables

**Table 1:** Baseline characteristics of children with chronic disease by nutritional status via body mass index (*n* = 78).

Characteristics	Total	Obesity	Undernutrition	Eutrophic	*p*-Value
	Mean ± SD	Mean ± SD	Mean ± SD	Mean ± SD	
*n*	78	24	30	24	
Female (%)	43 (55)	15 (62.5)	17 (56.7)	11 (45.8)	0.472
Age (years)	9.6 ± 4.8	11 ± 4	7 ± 5	10 ± 5	0.003 *
Age-for-50° Height (months)	115 ± 93	142 ± 62	72 ± 70	142 ± 122	0.005 *
Children (age in years)	6 ± 3	7 ± 3	4 ± 3	7 ± 3	0.026 *
Adolescent age in years)	13 ± 2	13 ± 2	13 ± 2	14 ± 2	0.363
Time of chronic disease (months)	66 ± 47	55 ± 38	65 ± 53	77 ± 47	0.282
Weight-for-age (kg)	38 ± 26	63 ± 24	18 ± 12	38 ± 18	0.000 *
Height-for-age (cm)	131 ± 31	147 ± 21	112 ± 30	139 ± 28	0.000 *
Height-for-age Z-score	−0.76 ± 1.5	−0.7 ± 1.3	−1.6 ± 1.6	−0.4 ± 1.2	0.000 *
Weight-for-Height Z-score	0.2 ± 2.1	2.5 ± 1.5	−1.7 ± 1.1	0.3 ± 1.2	0.000 *
Body mass index(kg/cm^2^)	19 ± 7.2	28 ± 5	13 ± 1.4	18 ± 2.8	0.000 *
Triceps skinfold (mm)	13 ± 9	24 ± 7	6 ± 2	11 ± 1	0.000 *
Muscle mass by anthropometry (Kg)	28 ± 15	40 ± 15	16 ± 9	29 ± 12	0.000 *
Fat mass by anthropometry (Kg)	11 ±11	23 ± 11	2 ± 2	9 ± 7	0.000 *
Muscle mass by BIA	30 ± 16	41 ± 15	19 ± 18	29 ± 13	0.000 *
Fat mass by BIA	12 ± 11	22 ± 12	4 ± 4	9 ± 6	0.000 *
Bone conduction speed	1923 ± 88	1931 ± 71	1909 ± 99	1933 ± 90	0.561
Blood Analytic					
Prealbumin (mg/dL)	22 ± 0.6	23 ± 6	21 ± 6	21 ± 6	0.185
Albumin (g/dL)	4.3 ± 0.3	4.2 ± 0.3	4.3 ± 0.3	4.3 ± 0.3	0.533
Transferrin (mg/dL)	254 ± 34	258 ± 27	257 ± 40	248 ± 33	0.538
Leucocytes (cell/mm^3^)	7465 ± 2239	7025 ± 2373	8344 ± 2349	6806 ± 1593	0.020 *
Lymphocytes (cell/mm^3^)	3080 ± 1432	2887 ± 312	3606 ± 1641	2615 ± 719	0.028 *
Total cholesterol (mg/dL)	174 ± 38	161 ± 29	174 ± 40	187 ± 40	0.072
LDL-cholesterol (mg/dL)	104 ± 30	94 ± 31	104 ± 28	111 ± 32	0.187
Blood urea nitrogen	30 ± 9	30 ± 6	28 ± 8	33 ± 11	0.163
Calcium (mg/dL)	10 ± 0.5	9.8 ± 0.5	10 ± 0.5	10 ± 0.4	0.129
Phosphorus (mg/dL)	4.8 ± 0.6	4.7 ± 0.6	4.9 ± 0.6	4.6 ± 0.5	0.233
IGF-1 (ng/mL)	212 ± 136	264 ± 119	149 ± 115	241 ± 152	0.004 *
IGFBP3 (μg/mL)	2.8 ± 0.8	3.2 ± 0.8	2.4 ± 0.8	2.9 ± 0.8	0.004 *
Iron (µg/dL)	78.6 ± 31	80.8 ± 20	75.7 ± 40	80.4 ± 28	0.806
C-reactive protein (U/L)	3.8 ± 1.6	3.8 ± 1.3	3.6 ± 0.9	3.9 ± 2.4	0.795
Erythrocyte sedimentation rate (mm/h)	15 ± 8	15 ± 9	15 ± 8	14 ± 8	0.943
Gamma-glutamyl transferase	16 ± 24	15 ± 6	18 ± 38	13 ± 4.6	0.000 *
Alanine aminotransferase	28 ± 10	25 ± 8	34 ± 11	24 ± 7	0.725
Zinc (µg/dL)	87 ± 12	87 ± 12	85 ± 13	88 ± 13	0.761
Copper (µg/dL)	118 ± 29	119 ± 23	114 ± 35	122 ± 3	0.622
Copper/Zinc ratio	1.4 ± 0.4	1.4 ± 0.2	1.4 ± 0.2	1.4 ± 0.4	0.845
Zinc/Copper ratio	0.8 ± 0.6	0.7 ± 1.4	0.9 ± 0.9	0.8 ± 0.2	0.480
Hypocupremia cases (%)	4 (5)	1 (4)	3 (10)	0	0.762
Hypercupremia cases (%)	13 (17)	4 (17)	5 (17)	4 (17)	0.762
Hypozincemia cases (%)	5 (6)	2 (8)	2 (6)	1 (4)	0.840
Copper/Zinc ratio > 1 (%)	68 (87)	23 (99)	25 (83)	20 (83)	0.318
Copper/Zinc ratio > 2 (%)	4 (5)	0	2 (7)	2 (8)	0.382
Zinc/Copper ratio > 4 (%)	1 (1)	0	1 (3)	0	0.449
**Prospective Dietary Survey**					
Dietary Zn intake (%DRI)	69 ± 35	81 ± 40	60 ± 30	67 ± 35	0.110
Dietary Zn intake (mg/day)	10 ± 5	12 ± 6	9 ± 4	10 ± 5	0.109
Protein (%DRI)	276 ± 176	248 ± 140	307 ± 222	266 ± 140	0.453
Carbohydrates (%DRI)	79 ± 35	81 ± 50	79 ± 26	77 ± 27	0.934
Total lipids(%DRI)	111 ± 40	100 ± 28	108 ± 43	126 ± 45	0.078
Cholesterol (%DRI)	265 ± 131	317 ± 113	231 ± 120	257 ± 149	0.053
Vitamin A (%DRI)	248 ± 960	87 ±78	165 ± 135	508 ± 1706	0.268
Folic acid (%DRI)	167 ± 86	189 ± 83	133 ± 69	187 ± 96	0.024 *
Fiber (%DRI)	16 ± 7	17 ± 7	15 ± 8	18 ± 5	0.319
Kilocalories (%DRI)	94 ± 24	87 ± 23	96 ± 25	98 ± 23	0.276
Dietary Zn deficiency (%)	53 (68)	14 (58)	22 (73)	17 (71)	0.388

Abbreviations: LDL: Low-density lipoprotein. BIA: bioelectrical impedance analysis. % DRI: percentage of dietary reference intake. NV: normal values. * *p*-value < 0.05.

**Table 2:** List of patients with abnormal serum copper and zinc levels, high copper/zinc and zinc/copper ratios, and deficient zinc intake (*n* = 21).

Gender by Group	Age (Years)	Serum Zn (µg/dL)	Serum Cu (µg/dL)	Cu/Zn Ratio	Zn/Cu Ratio	Dietary Zn Intake (%DRI)	CRP	ESR
Obesity								
Female	2	93	159 **	1.7 ^†^	0.6	54 ^‡^	3.2	19 ^##^
Male	5	92	142 **	1.5 ^†^	0.6	111	3.1	14
Female	5	116	153 **	1.3 ^†^	0.8	69 ^‡^	3.4	26 ^##^
Female	12	74	65 ***	0.9	1.1	77 ^‡^	3.1	14
Male	12	96	150 **	1.6 ^†^	0.6	118	3.1	16 ^##^
Female	13	69 *	80	1.2 ^†^	0.9	74 ^‡^	3.5	37 ^##^
Male	13	61 *	108	1.8 ^†^	0.6	74 ^‡^	3.75	36 ^##^
Undernutrition								
Male	2	66 *	87	1.3 ^†^	0.8	20 ^‡^	5.1 ^#^	24 ^##^
Male	2	117	20 ***	0.2	5.8 ^††^	18 ^‡^	3.2	14
Female	2	82	153 **	1.9 ^†^	0.5	140	6.1 ^#^	10
Male	3	85	150 **	1.8 ^†^	0.6	72 ^‡^	3.7	18 ^##^
Male	4	76	143 **	1.9 ^†^	0.5	96	3.7	30 ^##^
Male	6	79	188 **	2.4 ^†^	0.4	20 ^‡^	2.9	26 ^##^
Male	9	94	189 **	2 ^†^	0.5	47 ^‡^	6.4 ^#^	21 ^##^
Female	11	64 *	125	1.9 ^†^	0.5	52 ^‡^	3.5	25 ^##^
Male	15	82	65 ***	0.8	1.3	50 ^‡^	3.7	11
Female	16	72	69 ***	0.9	1	51 ^‡^	3.3	10
Eutrophic								
Male	2	77	175 **	2.3 ^†^	0.4	31 ^‡^	3.1	26 ^##^
Male	2	85	163 **	1.9 ^†^	0.5	24 ^‡^	10.7 ^#^	22 ^##^
Male	3	68 *	160 **	2.3 ^†^	0.7	76 ^‡^	3.1	26 ^##^
Male	5	106	194 **	1.8 ^†^	0.5	58 ^‡^	3.1	14

Legend: * Hypozincemia: serum Zn levels below 70 µg/dL in children under 10 years of age in both sexes and in females older than 10 years and below 74 µg/dL in males older than 10 years [42]. ** Hypercupremia: >140 µg/dL and *** Hypocupremia: <70 µg/dL [37]. ^†^ Cu/Zn ratio < 1 [39]. ^††^ Zn/Cu ratio > 4.0 [40]. **^‡^** Deficient Zn intake < 80% DRI: percentage of dietary reference intake. ^#^ CRP: C-reactive protein > 4 U/L. ^##^ ESR: erythrocyte sedimentation rate in women >20 mm/h and men >15 mm/h.

**Table 3:** Differences between participants with chronic diseases (*n* = 78).

Characteristics	Male	Female	*p*-Value
Children age (years)	6 ± 3	6 ± 3	0.851
Adolescent age (years)	13 ± 2	14 ± 2	0.250
Serum copper level (µg/dL)	123 ± 37	113 ± 21	0.138
Copper/zinc ratio	1.4 ± 0.5	1.3 ± 0.3	0.108
Zinc/copper ratio	0.9 ± 0.9	0.8 ± 0.1	0.501
Serum zinc concentration (µg/dL)	87 ± 12	87 ± 13	0.967
Dietary zinc intake (%Dietary Reference Intake)	67 ± 29	70 ± 41	0.783
Dietary zinc intake (mg/day)	10 ± 4	10 ± 6	0.773
**Age Group**	**Children**	**Adolescent**	
Serum copper level (µg/dL)	128 ± 32	106 ± 20	0.001 *
Copper/zinc ratio	1.5 ± 0.4	1.2 ± 0.3	0.004 *
Zinc/copper ratio	0.8 ± 0.8	0.8 ± 0.6	0.953
Serum zinc concentration (µg/dL)	88 ± 12	85 ± 13	0.393
Dietary zinc intake (%Dietary Reference Intake)	74 ± 44	63 ± 23	0.206
Dietary zinc intake (mg/day)	11 ± 6	9 ± 3	0.201
**C-Reactive Protein**	**Normal**	**High**	
Serum copper level (µg/dL)	115 ± 25	136 ± 32	0.036 *
Copper/zinc ratio	1.3 ± 0.3	1.6 ± 0.3	0.047 *
Zinc/copper ratio	0.8 ± 0.2	0.6 ± 0.1	0.029 *
Serum zinc concentration (µg/dL)	87 ± 12	84 ± 8	0.604
Dietary zinc intake (%Dietary Reference Intake)	70 ± 36	70 ± 39	0.992
Dietary zinc intake (mg/day)	10 ± 5	10 ± 6	0.996
**Erythrocyte Sedimentation Rate**	**Normal**	**High**	
Serum copper level (µg/dL)	110 ± 27	136 ± 31	0.001 *
Copper/zinc ratio	1.2 ± 0.3	1.7 ± 0.4	0.000 *
Zinc/copper ratio	0.9 ± 0.7	0.6 ± 0.1	0.084
Serum zinc concentration (µg/dL)	89 ± 11.9	81 ± 13	0.011 *
Dietary zinc intake (%Dietary Reference Intake)	69 ± 29	66 ± 37	0.707
Dietary zinc intake (mg/day)	10 ± 4	10 ± 5	0.698

* Correlation is significant at the 0.05 level (two-tailed). Results are given in means and standard deviation.

**Table 4:** Odds Ratio in the whole series (*n* = 78).

	Fisher’s Exact Test	Odds Ratio	95% Confidence Interval		Cochran’s	Mantel–Haensze
			Lower	Upper		
Abnormal copper levels						
Males	0.016	3.965	1.237	12.78	0.016	0.034
Children	0.031	3.59	1.050	12.247	0.034	0.067
Children < 5 years	0.005	5.889	1.759	19.712	0.002	0.007
Age-for-50° Height	0.010	4.381	1.418	13.536	0.007	0.018
Low weight-for-age	0.014	4.154	1.338	12.891	0.010	0.025
Low folic acid intake	0.011	7.917	1.662	37.707	0.004	0.014
High erythrocyte sedimentation rate	0.013	4.950	1.577	15.538	0.005	0.014
Leukocytosis	0.013	8.056	1.847	35.132	0.003	0.013
Hypercupremia						
Males	0.012	5.333	1.337	21.276	0.011	0.026
Children	0.022	14	2.405	81.486	0.002	0.006
Children < 5 years	0.004	7.259	1.983	26.580	0.001	0.004
Age-for-50° Height	0.003	6.891	1.867	25.436	0.002	0.005
High vitamin A intake	0.017	4.607	1.340	15.840	0.014	0.033
High erythrocyte sedimentation rate	0.000	10.8	2.929	39.828	0.000	0.001

**Table 5:** Association between bone conduction speed via bioelectrical impedance analysis with anthropometric assessment (*n* = 78).

	Total Series		Obesity		Undernutrition		Eutrophic	
	*r*	*p*-Value	*r*	*p*-Value	*r*	*p*-Value	*r*	*p*-Value
Age (months)	0.773 **	0.000	0.761 **	0.000	0.732 **	0.000	0.876 **	0.000
Age-for-50° height	0.638 **	0.000	0.539 *	0.012	0.799 **	0.000	0.639 **	0.001
Weight-for-age	0.525 **	0.000	0.589 **	0.005	0.786 **	0.000	0.834 **	0.000
Height-for-age	0.742 **	0.000	0.700 **	0.000	0.791 **	0.000	0.834 **	0.000
Weight-for-height	0.287 *	0.016	-	-	-	-	0.576 **	0.004
Body mass index	0.261 *	0.029	-	-	-	-	0.626 **	0.001
Muscle mass by A. (Kg)	0.622 **	0.000	0.653 **	0.001	0.765 **	0.000	0.889 **	0.000
Fat mass by A. (Kg)	0.354 **	0.003	0.453 *	0.039	0.737 **	0.000	0.598 **	0.003
Muscle mass by BIA	0.490 **	0.000	0.492 *	0.023	-	-	0.871 **	0.000
Fat mass by BIA	0.330 **	0.008	0.572 **	0.007	-	-	0.561 **	0.005

Legend: A: Anthropometry. BIA: bioelectrical impedance analysis. * *p* < 0.05. ** *p* < 0.01 (2-tailed).

**Table 6:** Multiple linear regression between serum copper and Cu/Zn and Zn/Cu ratios with nutritional parameters studied in the whole series (*n* = 78).

Serum Copper	Cu/Zn Ratio	Zn/Cu Ratio
*r* = 0.493 Height-for age *p* = 0.000 Height-for-age Z score *p* = 0.024 Triceps skinfold *p* = 0.032	*r* = 0.387 Height-for age *p* = 0.000 Triceps skinfold *p* = 0.045	*r* = 0.356 Height-for age *p* = 0.000 Triceps skinfold *p* = 0.012
*r* = 0.295 BCS absolute value *p* = 0.000	*r* = 0.260 BCS absolute value *p* = 0.000	*r* = 0.224 BCS absolute value *p* = 0.000
*r* = 0.230 Fiber %DRI *p* = 0.000 Magnesium %DRI *p* = 0.016 Vitamin B6 %DRI *p* = 0.021	*r* = 0.054 Fiber %DRI *p* = 0.042	*r* = 0.534 Vitamin B1 %DRI *p* = 0.000 Vitamin B2 %DRI *p* = 0.002 Vitamin B6 %DRI *p* = 0.034
*r* = 0.409 Prealbumin *p* = 0.000 GGT *p* = 0.002 Cardiovascular risk index *p* = 0.005	*r* = 0.401 Prealbumin *p* = 0.003 GGT *p* = 0.002 Total bilirubin *p* = 0.007	*r* = 0.940 GGT *p* = 0.000 ALT *p* = 0.000 Prealbumin *p* = 0.012 Total bilirubin *p* = 0.024
*r* = 0.250 IGF-1 *p* = 0.000	*r* = 0.218 IGF-1 *p* = 0.000	*r* = 0.157 IGF-1 *p* = 0.001
*r* = 0.400 Leucocytes *p* = 0.049 Hemoglobin *p* = 0.002 MCHC *p* = 0.028 Neutrophils *p* = 0.036	*r* = 0.435 Hemoglobin *p* = 0.000 Neutrophils *p* = 0.000	*r* = 0.350 ESR *p* = 0.000 MCV *p* = 0.016 CRP *p* = 0.036
*r* = 0.365 IgG4 *p* = 0.000 IgG1 *p* = 0.012 T-lymphocytes CD4 *p* = 0.041	*r* = 0.207 IgG3 *p* = 0.014 IgG4 *p* = 0.019	*r* = 0.075 IgG4 *p* = 0.045
	*r* = 0.056 Time of illness *p* = 0.036	

Correlation is significant at the 0.05 level (two-tailed). Legend: BCS: Bone conduction speed. %DRI: Percentage dietary reference intake. GGT: Gamma-glutamyl transferase. ALT: Alanine aminotransferase. IGF-1: Insulin-like growth factor-1. MCHC: Mean corpuscular hemoglobin concentration. MCV: Mean corpuscular volume. ESR: Erythrocyte sedimentation rate. CRP: C-reactive protein. Ig: Immunoglobulin.

**Table 7:** Multiple linear regression between serum copper and Cu/Zn and Zn/Cu ratios with nutritional parameters studied by nutritional status via BMI (*n* = 78).

Obesity			Undernutrition			Eutrophic		
Serum Copper	Cu/Zn Ratio	Zn/Cu Ratio	Serum Copper	Cu/Zn Ratio	Zn/Cu Ratio	Serum Copper	Cu/Zn Ratio	Zn/Cu Ratio
*r* = 0.591 Height-for age *p* = 0.000 Height-for-age Z score *p* = 0.013	*r* = 0.418 Height-for age *p* = 0.005 Height-for-age Z score *p* = 0.047		*r* = 0.686 Kg mass muscular *p* = 0.001 Suprailiac skinfold Z-score *p* = 0.005 Waist/hip ratio *p* = 0.024	*r* = 0.318 Wrist perimeter *p* = 0.010	*r* = 0.545 Kg mass muscular *p* = 0.005 Hip perimeter. *p* = 0.030	*r* = 0.724 Waist/Height ratio *p* = 0.0001 Arm muscular area *p* = 0.0002	*r* = 0.747 Height-for age *p* = 0.000 Wrist perimeter *p* = 0.000 Body mass index Z-score *p* = 0.031	*r* = 0.400 Kg mass muscular *p* = 0.002
*r* = 0.421 BCS absolute value *p* = 0.002	*r* = 0.316 BCS absolute value *p* = 0.010	*r* = 0.214 BCS absolute value *p* = 0.040				*r* = 0.582 BCS absolute value *p* = 0.000	*r* = 0.494 BCS absolute value *p* = 0.000	*r* = 0.463 BCS absolute value *p* = 0.000
*r* = 0.672 Fiber %DRI *p* = 0.012 Folic acid %DRI *p* = 0.002 Magnesium %DRI *p* = 0.001	*r* = 0.384 Folic acid %DRI *p* = 0.001	*r* = 0.409 Folic acid %DRI *p* = 0.001	*r* = 0.364 Vitamin B1 %DRI *p* = 0.002 Fiber %DRI *p* = 0.017	*r* = 0.319 Vitamin B1 %DRI *p* = 0.003 Fiber %DRI *p* = 0.035	*r* = 0.917 Vitamin B1 %DRI *p* = 0.000 Vitamin B6 %DRI *p* = 0.000 Cholesterol %DRI *p* = 0.029	*r* = 0.506 Carbohydrates %DRI *p* = 0.006 Fiber %DRI *p* = 0.006 Protein %DRI *p* = 0.042	*r* = 0.362 Vitamin B1 %DRI *p* = 0.003 Magnesium %DRI *p* = 0.035	*r* = 0.166 Vitamin B1 %DRI *p* = 0.048
*r* = 0.399 Creatinine *p* = 0.012	*r* = 0.416 BUN *p* = 0.009	*r* = 0.426 BUN *p* = 0.008	*r* = 0.363 Transferrin saturation index *p* = 0.002	*r* = 0.602 Prealbumin *p* = 0.000 Cholesterol *p* = 0.029 Alkaline phosphatase *p* = 0.043	*r* = 0.990 GGT *p* = 0.000 Prealbumin *p* = 0.000 BUN *p* = 0.015	*r* = 0.618 Total bilirubin *p* = 0.000 Transferrin *p* = 0.003	*r* = 0.518 Total bilirubin *p* = 0.004 Transferrin *p* = 0.010	*r* = 0.511 Total bilirubin *p* = 0.001 Transferrin *p* = 0.044
*r* = 0.512 Phosphorus *p* = 0.027 IGF-1 *p* = 0.028	*r* = 0.241 Phosphorus *p* = 0.017	*r* = 0.213 Phosphorus *p* = 0.027	*r* = 0.307 IGF-1 *p* = 0.006	*r* = 0.308 IGF-1 *p* = 0.006	*r* = 0.284 IGF-1 *p* = 0.009	*r* = 0.350 IGF-1 *p* = 0.012	*r* = 0.610 IGF-1 *p* = 0.002 Phosphorus *p* = 0.016	*r* = 0.454 IGF-1 *p* = 0.008 IGBP3 *p* = 0.039
	*r* = 0.242 Hemoglobin *p* = 0.032		*r* = 0.398 Neutrophils *p* = 0.002	*r* = 0.569 Neutrophils *p* = 0.002 MCV *p* = 0.030	*r* = 0.409 Neutrophils *p* = 0.007 Eosinophils *p* = 0.046	*r* = 0.726 ESR *p* = 0.000 MCV *p* = 0.008 CRP *p* = 0.024	*r* = 0.850 ESR *p* = 0.000 Leucocytes *p* = 0.000 MCV *p* = 0.007 Basophils *p* = 0.015	*r* = 0.656 ESR *p* = 0.003 Leucocytes *p* = 0.009 CRP *p* = 0.023
	*r* = 0.882 IgG3 *p* = 0.000 IgG2 *p* = 0.000 IgG4 *p* = 0.006		*r* = 0.393 Lymphocytes CD16 + 56 *p* = 0.003 IgA *p* = 0.023	*r* = 0.382 Lymphocytes CD16 + 56 *p* = 0.005 IgA *p* = 0.020		*r* = 0.573 Complement C4 *p* = 0.000	*r* = 0.493 IgG2 *p* = 0.016 Complement C3 *p* = 0.024	*r* = 0.443 IgG2 *p* = 0.012 IgG1 *p* = 0.046

Legend: BCS: Bone conduction speed. %DRI: Percentage dietary reference intake. GGT: Gamma-glutamyl transferase. BUN: Blood urea nitrogen. IGF-1: Insulin-like growth factor-1. IGFBP3: insulin-like growth factor-binding protein 3. MCV: Mean corpuscular volume. ESR: Erythrocyte sedimentation rate. CRP: C-reactive protein. Ig: Immunoglobulin. CD16 + CD56+ Natural Killer cells.

## Abbreviations

CF	Cystic Fibrosis
BIA	Bioelectrical impedance analysis
BCS	Bone conduction speed
%DRI	Dietary Reference Intake
Cu/Zn	Copper/Zinc
Zn/Cu	Zinc/Copper
CRP	C-reactive protein
ESR	Erythrocyte sedimentation rate
IGF-1	Insulin-like growth factor-1
IGFBP3	Insulin-like growth factor-binding protein 3
OR	Odds ratio
GGT	Gamma-glutamyl transferase
ALT	Alanine aminotransferase
IDA	Iron deficiency anemia
ADHD	Attention deficit/hyperactivity disorder
ZICD	Zinc-induced Cu deficiency
MT	Metallothioneins
CDM	Copper deficiency myelopathy
BUN	Blood urea nitrogen
ASD	Autism spectrum disorder
